# The Stethoscope

**Published:** 1854-11

**Authors:** 


The Stethoscope, (Virginia,) comments at large upon the proceedings of the N. Y. State Medical Society last winter, relative to the censorship of colleges. The Stethoscope approves this action, and has a high opinion of the good results likely to obtain from a system of supervision by the State Society over the schools of the state. It also censures strongly the course of the University School, (Fourteenth St.,) in refusing this supervision. To all which we agree. H.

